# Path Integral Monte Carlo Simulation on Molecular Systems with Multiple Electronic Degrees of Freedom

**DOI:** 10.1021/acs.jctc.4c01717

**Published:** 2025-04-29

**Authors:** Michael Hütter, Milan Ončák

**Affiliations:** Institut für Ionenphysik und Angewandte Physik, Universität Innsbruck, 6020 Innsbruck, Austria

## Abstract

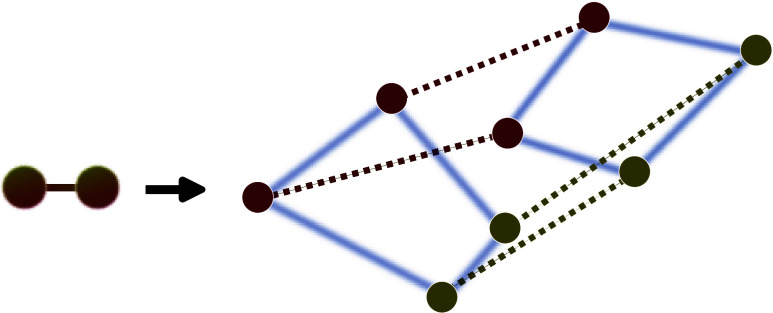

We present an imaginary time path-integral formalism for molecular systems including nuclear and electronic degrees of freedom based on the previous work of [SchmidtJ. R.; TullyJ. C.J. Chem. Phys.2007, 127, 09410317824728 10.1063/1.2757170]. To sample the resulting path integral expression efficiently, a path integral Monte Carlo scheme is proposed, allowing the computation of finite temperature equilibrium properties of molecular systems including multiple low-lying electronic states directly from ab initio potential energy surfaces. Finally, we show how this generalized approach in combination with the Monte Carlo scheme can reproduce exact results for a simple model system including nonadiabatic couplings as well as thermodynamic equilibrium properties of H_2_ and C_2_. Our implementation of the algorithm is available as an open-source code.

## Introduction

Path integral (PI) simulations based on Feynman’s imaginary time PI^1^ give numerically exact results with respect to quantum Boltzmann statistics within the canonical ensemble, making them a powerful method to compute finite temperature properties of molecules^2−6^ or strongly correlated quantum liquids like ultracold helium.^7,8^ The general idea behind these PI simulations is to define a classical isomorphic system whose statistical behavior is identical to the original quantum system. This is typically achieved by time discretizing the Wick-rotated PI into *P* segments, also known as beads, resulting in a partition function for *P* × *N* classical particles which, on average, resemble the statistical behavior of *N* quantum particles in thermal equilibrium. However, for nearly all applications, it is assumed that nuclear motion takes place on a single potential energy surface (PES),^9^ typically associated with the electronic ground state. This necessitates a formulation where in addition to the continuous nuclear degrees of freedom, the discrete electronic states are also incorporated into the partition function as nonadiabatic effects between multiple electronic states are fundamental to numerous processes in physics, chemistry, and biology, with applications ranging from spectroscopy and surface chemistry to photosynthesis.^10−12^

In this context, several approaches to finding a generalized nonadiabatic expression for the PI have been proposed. Most notably, Schwieters and Voth^13^ have derived one in the diabatic basis and applied it to quantum transition state theory to calculate rate coefficients. They also showed that, in the limit of well-separated adiabats, only the ground state contributes to the total partition function and thus reduces to the well-known path integral expression for a single PES. Further, Alexander derived an identical expression and showed that it can reproduce exact quantum results for a simple model system.^14^ He also showed that in the case of adiabatic coupling, the partition function reduces to a simple sum of partition functions, one for each PES. Nonadiabatic effects are subsequently introduced by defining a thermally averaged mean field potential, treating diabatic states in an average way. However, this results in many-body potential energy surfaces with no classical analogs that are thus difficult to interpret. Schmidt and Tully^15^ addressed this problem by introducing an expression for the partition function where the beads can individually move and make transitions among various adiabatic PESs and nonadiabatic effects are introduced from neighboring beads being on different PESs, resulting in an energy penalty which arises via an additional pseudopotential calculated from diabatic states. Just like in the adiabatic limit, this comes with the advantage of mapping onto a classical isomorphic system and thus easy interpretation.

Further, several nonadiabatic PI molecular dynamics (PIMD) approaches based on a semiclassical mapping protocol to derive an exact PI representation of the canonical partition function, including multiple electronic states, have been presented.^16−20^ While these works mainly focus on the computation of dynamics via quantum time correlation functions, they can also provide equilibrium observables. In this approach, special care is needed to maintain the desired temperature, and all beads need to be coupled to a thermostat.^2^ Additionally, open-chain PI models have also been proposed for the computation of two-state time correlation functions.^21^

Here, we present a general path integral expression for molecular systems including nuclear and electronic degrees of freedom similar to the one of Schmidt and Tully and propose a Path Integral Monte Carlo (PIMC) scheme that can be used to sample this path integral expression efficiently. Subsequently, we show that exact results can be reproduced for a simple model system composed of harmonic adiabats with off-diagonal Gaussian-type couplings that mimic nonadiabatic effects. Finally, we use the outlined method to compute equilibrium properties of two molecular systems, namely H_2_ and C_2_.

## Methods

### Path Integrals

As usual, the quantum partition function is given as the trace of the canonical density matrix 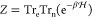
1with  being the Hamiltonian, β = 1/*k*_b_*T* the inverse temperature, and Tr_e_ and Tr_n_ should indicate explicitly that both the electronic and nuclear degrees of freedom are traced out. A PI expression of this partition function can be obtained from the Wick-rotated Feynman PI in imaginary periodic time βℏ
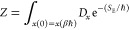
2with *S*_E_ being the Euclidean action and *x*(0) = *x*(βℏ) indicating that the integration is performed over all closed paths. To find a computationally tractable expression of eq 2, it can be time discretized into *P* segments known as beads. This gives
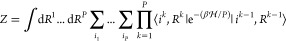
3with |*i*,*R*⟩ being the product of electronic wave function *i* and nuclear coordinate *R*. Note that the cyclic condition is implicitly assumed, meaning that if *k* = *P*, *R*^*k*+1^ ≡ *R*^1^. The evaluation of the quantum partition function as given by eq 3 can be found elsewhere^9^ and results in
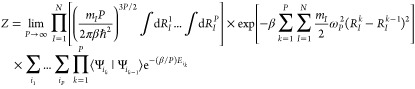
4with *m*_*I*_ being the mass of particle *I*, , Ψ_*i*_*k*__ is the electronic wave function and *E*_*i*_*k*__ the electronic energy of bead *k* in an electronic state *i*. Note that eq 4 gives exactly the general form of the partition function we need to treat molecular systems including multiple PESs and possibly also nonadiabatic effects. With this, the classical isomorphic Hamiltonian  is given by
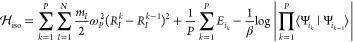
5

The first term of eq 5 as usual defines a kinetic spring term that acts between different beads of the same atom and represents the quantum kinetic energy of the particles. The second term defines the potential, where each bead can move and make transitions individually among the different adiabatic PESs *i*. Note that the index *i*_*k*_ results from the summation in eq 4. The third term can be seen as a temperature-dependent pseudopotential
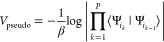
6that has a very distinct role as it introduces the nonadiabatic effects.^15^ This can be seen easily as in the case of adiabatic PESs we have ⟨Ψ_*i*_*k*__|Ψ_*i*_*j*__⟩= δ_*i*_*k*_,*i*_*j*__. Thus, the overlap terms in eq 6 would evaluate to zero, making the total expression approach infinity, if two neighboring beads are located on different PESs. Therefore, in the adiabatic limit, all beads need to be located on the same PES. If nonadiabatic couplings should be considered, the PESs can be expressed in the diabatic basis, thus resulting in potentially small nonvanishing overlap terms ⟨Ψ_*i*_*k*__|Ψ_*i*_*k*–1__⟩. This will introduce a type of energy penalty via the pseudopotential *V*_pseudo_ into the total action of the system if neighboring beads are located on different PESs. So to treat nonadiabatic effects, in principle, for each time step of the MC simulation, it will be necessary to diagonalize *P* distinct *n* × *n* diabatic matrices with *n* being the number of diabatic states. Finally, it is worth noting that during the derivation of eq 4 the so-called primitive approximation to the PI action was applied. This means that in principle the noncommutativity of the kinetic and potential energy operators is ignored, resulting in errors on the order of (β/*P*)^2^ that can be made arbitrarily small by increasing the number of beads *P*, however with it increasing the computational cost of the simulation.

### Monte Carlo Algorithm

To sample the classical isomorphic Hamiltonian as given by eq 5, a Markov-Chain Monte Carlo algorithm (MCMC) is used. More specifically, the Metropolis-Hastings criterion is used to implement the MCMC algorithm. This algorithm was chosen as it is very flexible in sampling from generic probability distributions by generating a Markov chain whose stationary distribution π(*X*) converges to the desired target distribution. Here, the target distribution is given by the Boltzmann distribution of the classical isomorphic Hamiltonian, meaning that .

In practice, the algorithm can be implemented by proposing a transition from configuration *X* to *X*′ according to some conditional proposal distribution *W*(*X*,*X*′) and subsequently accepting this transition with a probability of
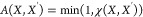
7where
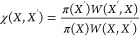
8Note that as long as *W*(*X*,*X*′) is a symmetric probability distribution, meaning that the proposed move *X* → *X*′ is reversible, the acceptance probability χ will simplify to χ = π(*X*′)/π(*X*) and thus the probability to accept a move in a PIMC simulation is given by
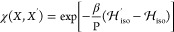
9To achieve optimal convergence and save computational resources, it is important to generate samples *X*′ from the proposal distribution *W*(*X*,*X*′) that represent the configuration space of possible bead configurations (as described by the classical isomorphic Hamiltonian) as closely as possible while keeping the correlation between successive samples as small as possible. This means that the proposed moves should explore all possible configurations of the beads efficiently while keeping high acceptance rates. This can be challenging in MC simulations as in principle there are two opposite effects. On the one hand, single-bead moves, like changing the position of one individual bead, typically result in a high acceptance probability. However, they also involve recomputing the potential and kinetic action of the whole chain after each step and propose only small changes to the current state, resulting in a slow exploration of the configuration space and high autocorrelation between subsequent samples. This means that single-bead moves in general can become very inefficient for a larger number of beads *P*. Multibead moves, on the other hand, are computationally much more efficient, as in principle the free particle distribution can be sampled directly^2^ and thus only the change in potential energy can be used to build the acceptance probability. However, this approach, known as staging, is not applicable to the entire chain of beads, like in PI molecular dynamics,^2^ as these moves would result in a too low acceptance probability for most practical cases. Therefore, we use a combination of multiple moves to achieve efficient sampling of both the nuclear and electronic degrees of freedom.

First, center of mass (CoM) moves are implemented to sample the potential action by uniformly translating the entire path of a single atom, leaving the kinetic action unchanged. This means that all beads corresponding to a randomly chosen atom are translated by a random vector  with
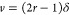
10Here , while its vector components *r*_*i*_ ∈ *U*([0,1)) are uniformly distributed, and  controls the acceptance ratio, as it scales *v* to be uniformly distributed in the interval [−δ, δ).

Second, as mentioned before, staging moves are only applied to a section of path between two fixed beads to keep the acceptance probability high. The length, and thus the acceptance probability, of this stage is controlled by a parameter α, where initially one bead *i* is chosen at random, and subsequently, a new section of path is generated between the two fixed beads *j* and *j* + α, where the kinetic action of the displaced path segment is sampled directly using the Lévy construct.^22^

Third, global bead excitations are implemented to change the electronic state of all beads simultaneously, as needed in the adiabatic limit, as here all beads need to be located on the same PES. Let *E*_c_ denote the current electronic state and Σ = {*E*_1_, ···, *E*_*n*_}\{*E*_c_} be the set of all possible electronic states excluding the current one. The global bead excitation move is then implemented by assigning a new electronic state *E*_new_ according to the following probability distribution
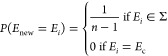
11meaning that each possible state, excluding the current one, is equally likely to be chosen.

Finally, to efficiently sample the pseudopotential, as needed in the diabatic limit, two additional MC moves are implemented. As discussed before, nonadiabatic effects are introduced via the temperature-dependent pseudopotential of eq 6 if two neighboring beads are located on different PESs. We can make use of this by introducing a MC move that sets a parameter ξ, controlling the fixed number of overlap terms that should be present in the pseudopotential *V*_pseudo_
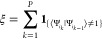
12where **1**_{_···_}_ is an indicator function that returns one if neighboring beads are located on different PESs. So the total expression is equal to the number of overlap terms that need to be computed, with ξ taking possible values of
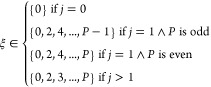
13Here, *P* is as usual the number of beads while *j* is the number of excited electronic states, so *j* = 0 corresponds to the case where only the ground state is considered. With a second move, this fixed number of overlap terms, as set by ξ, is propagated through the necklace of beads. This means that ξ is held constant while the electronic state of individual beads is changed. Practically, these two moves are combined to perform the so-called propagation of excitation (PoE) move by periodically setting a fixed number of overlap terms from uniformly sampling the possible values of ξ, as given by eq 13. Subsequently, these overlap terms are propagated through the necklace of beads and possible electronic states using a random walk, meaning that in each move, the electronic states of individual beads are changed in a way to be compatible with the current value of ξ. Note that in this random walk, the specific electronic configurations of the beads can be framed as microstates in a Potts-like model^23^ where we want to generate new microstates compatible with the microscopic total energy.

In total, this highly increases the acceptance probability as in principle, one overlap term is replaced by another one and thus the total change in the potential action is not as significant as if, for example, only the electronic state of one bead would be changed at random.

Finally, from the sampled configurations *X*_1_, ···, *X*_*n*_, it is easily possible to compute quantum equilibrium statistical averages as an ensemble average over the classical isomorphic system denoted by ⟨···⟩. For purely position-dependent operators , this gives
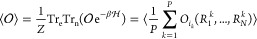
14where *O*_*i*_*k*__ should indicate the estimator for bead *k* in electronic state *i*. Estimates for operators that depend both on position and momenta can be computed in the usual way.^2^ For example, the energy estimator
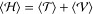
15can be computed using eq 14, as  is purely position-dependent, and the kinetic energy estimator given by
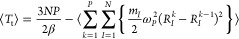
16or in its centroid virial form
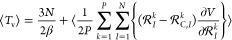
17with
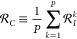
18Note that as usual, both ⟨*T*_t_⟩, known as the thermodynamic estimator, and ⟨*T*_v_⟩, known as the virial estimator, converge to the same mean, however ⟨*T*_v_⟩ typically reduces the variance significantly.^2^

To further obtain the heat capacity *C*_V_, the total energy was simply differentiated numerically with respect to the temperature
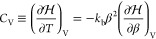
19However, as Monte Carlo estimates like the total energy  inherently suffer from variations, a direct computation of numerical gradients will greatly amplify these uncertainties, thus making the obtained heat capacities unreliable. To circumvent this, Tikhonov regularization^24^ based on a total-variation (TV) regularization term is used. The idea is to consider the derivative of  as an inverse problem, which is regularized using TV, while a data fidelity term (DF) penalizes the discrepancies between the predicted and observed data. Meaning that
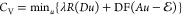
20with  being a sequence of energy measurements at different temperatures *T*_1_, ···, *T*_*n*_, *R* the TV regularization term, λ the regularization parameter that balances fidelity and smoothness, *D* a differentiation operator, DF the data fidelity term given by the square of the *L*^2^ norm, and *A* an antidifferentiation operator. For further details on specific choices of *R*, DF, *A* and *D*, see ref (25).

An open-source implementation of the above-described PIMC algorithm is freely available on GitHub,^26^ together with various sample input files such as the three-dimensional (3D) harmonic oscillator or H_2_. To further make the implementation as reusable and modular as possible, most of the code is written in Python while only performance-critical parts are either optimized using a Just-In-Time compiler or written in C.

## Applications

### Model System

**Figure 1:**
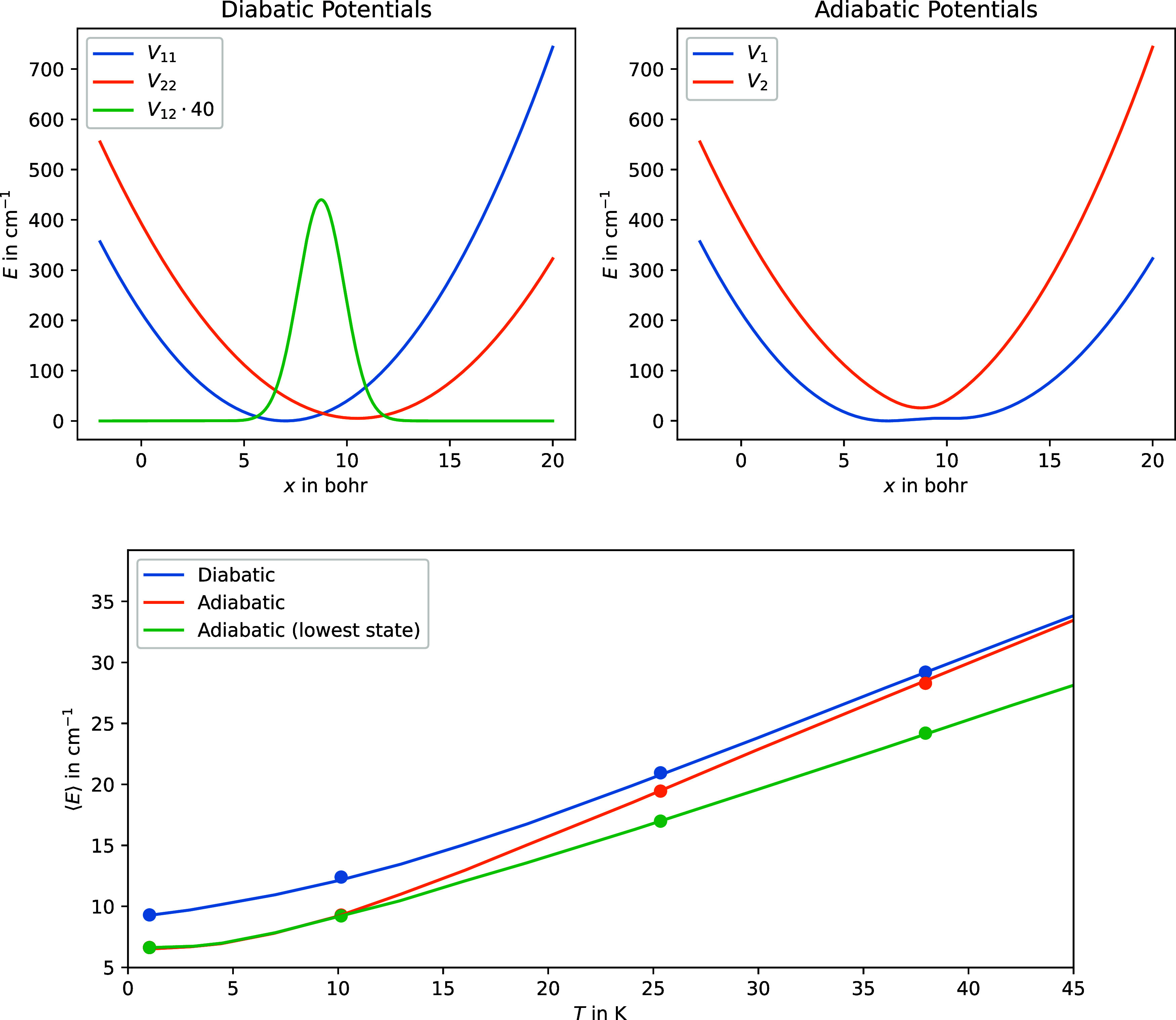
Upper two inserts show the diabatic potentials as given by eq 21 and the resulting adiabatic potentials of the model system, respectively. The lower inset shows the resulting expectation value of the energy estimator, as sampled via the PIMC code, as well as the exact energy, as a function of temperature, respectively. The continuous lines indicate the exact results and the points indicate the PIMC results.

To benchmark the outlined method in the spirit of Alexander’s work,^14^ we apply it to a simple model potential consisting of two harmonic diabats with an off-diagonal Gaussian diabatic coupling term as described by the following elements of the potential energy matrix
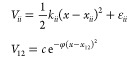
21where *i* = 1, 2 and *V*_12_ = *V*_21_. The numerical values for the parameters are summarized in Table 1, the potentials are depicted in Figure 1.

**Table 1:** Parameters to Define the Potential as Given by Equation 21^a^

parameter	value
*k*_11_	4 × 10^–5^
*k*_22_	3.2 × 10^–5^
*x*_11_	7
*x*_22_	10.5
ϵ_11_	0
ϵ_22_	2.2782 × 10^–5^
*c*	5 × 10^–5^
φ	0.4
*x*_12_	8.75
*m*	3.6743 × 10^3^

a All values are given in atomic units and were taken from ref (14).

Equation 21 provides an ideal benchmark potential as it can be seen as a simple model system for a diatomic molecule with two low-lying PESs. Further, as the off-diagonal Gaussian terms introduce some coupling between the two PESs, it is also well suited to investigate nonadiabatic effects that in real molecular systems can arise from the breakdown of the Born–Oppenheimer approximation.^27^

In the simulation, the quantum particle was discretized into 20 beads, and a total of 10^6^ MC sweeps were used. For each MC sweep, either a CoM, or a staging move, with a stage length of α = 12, followed by a PoE move was attempted. To further improve the sampling efficiency, for every fifth MC sweep, a global excitation of all beads simultaneously was attempted. In Figure 1, we show the computed expectation value for the energy estimator, given by eq 15, as a function of temperature, in comparison to the exact results as obtained via a standard sum-overstates approach. First, it is evident that the proposed PIMC algorithm can very well reproduce the exact results. Second, only considering the lowest adiabatic state becomes a worse approximation as the temperature increases. This is intuitively clear as populating higher electronic states should become more probable as the thermal energy in the system increases. Third, it also shows how considering nonadiabatic effects becomes crucial in regions with strong couplings, i.e., at low temperatures. Note that in Figure 1, the adiabatic results deviate from the diabatic ones in the low-temperature regime resulting from the fact that, at these temperatures, motion occurs mainly near the minimum of the potential curves, where the coupling between the two is most dominant (see the diabatic potential in Figure 1). However, at higher temperatures, motion can occur over a larger range of configurations and thus the region of nonadiabatic coupling becomes less significant, resulting in the convergence of the adiabatic and diabatic results at high temperatures.

### H_2_ Molecule

The H_2_ molecule is an example of one of the simplest molecular systems where only one electronic state needs to be accounted for and numerically exact results are still easily obtainable as its PES can be represented very well via a Morse potential. Therefore, the ^1^Σ_g_^+^ ground state PES was calculated in a range of 0.5 to 5 Å using Gaussian 16,^28^ employing a potential curve obtained through Full Configuration Interaction (FCI) with the aug-cc-pVDZ basis set and fitted to a Morse potential, the results are summarized in Table 2.

**Table 2:** Parameters of the Morse Potential, *V*(*r*) = *D*(1 – e^–*ϱ*(*r*–*r*_e_)^)^2^, as Obtained through Fitting the PES of H_2_ at the FCI/aug-cc-pVDZ Level of Theory

	value
*r*_e_ [Å]	0.763(2)
*ϱ* [Å^–1^]	2.02(2)
*D* [eV]	4.65(2)

For the MC simulation, the H_2_ molecule was discretized into 20 beads per H atom and run for 10^9^ MC sweeps. The stage length was set to α = 12 and for every MC sweep, either a staging or a CoM move was attempted. Note that as only one PES is present, no excitation moves need to be attempted. To compare the PIMC estimates to exact values, the Schrödinger equation was solved for a Morse potential as given in Table 2, which can be achieved analytically using a supersymmetric approach to quantum mechanics.^29^ Subsequently, expectation values for the equilibrium bond length *r*_0_, the internal energy *U*, and the heat capacity at constant pressure *c*_p_ were calculated using a standard statistical mechanics approach. A comparison of these estimates at 300 K reveals that within the variance of the MC algorithm, all estimates agree with the exact results, see Table 3. Note that to compute *c*_p_, Mayer’s relation was assumed, meaning that *c*_p_ = *c*_v_ + *R*.

**Table 3:** Comparison of the Equilibrium Bond Length *r*_0_, the Internal Energy *U*, and the Heat Capacity *c*_p_ as Calculated via the PIMC Algorithm and Analytically at 300 K (See Text for Details)

	calculated (PIMC)	analytical
*r*_0_ [Å]	0.786(12)	0.786
*U* [eV]	0.271(11)	0.276
*c*_p_ [J·mol^–1^·K^–1^]	29.1(14)	28.8

### C_2_ Molecule

Diatomic carbon has been a subject of extensive experimental and theoretical research^30^ as it can be found in various terrestrial and astrophysical sources.^31^ The molecule shows multiple low-lying PESs, and initial studies suggested that *a*^3^Π*_u_* is its ground electronic state.^30^ However, later *X*^1^Σ_g_^+^ is identified as the ground state.^32^ The two states show an energetic separation of roughly 700 cm^–1^ and spin–orbit corrections have been found to be insignificant.^33^ This complex electronic structure offers a challenge for existing PI methods to obtain accurate thermal properties, as both electronic states will be thermally accessible.

**Figure 2:**
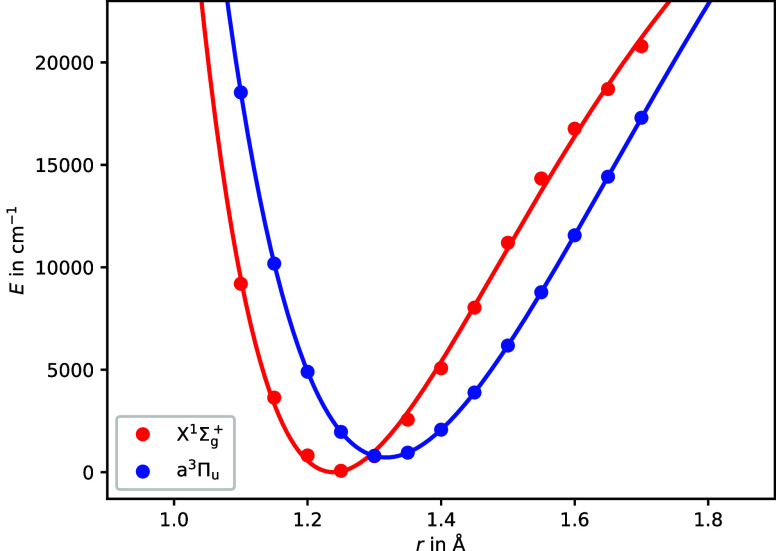
Potential energy surfaces of C_2_ calculated at a MRCI+Q(8,8)/aug-cc-pVQZ level of theory. The continuous lines indicate the Morse potential that was fitted to the calculated points.

The present approach can be used to treat such molecular systems and as an illustration, we computed the heat capacity of C_2_. To do so, the PESs were first calculated using Molpro 2012.1^34,35^ employing Multireference Configuration Interaction including Davidson correction, MRCI+Q(8,8)/aug-cc-pVQZ level of theory and subsequently fitted to a Morse potential, see Figure 2. This resulted in an energy separation between the minima of the two states of 638 cm^–1^. However, to minimize the effect of systematic errors introduced into the PIMC simulations due to inaccurate PESs separations, this energetic offset was as the only parameter adjusted manually to 716 cm^–1^ as measured experimentally.^36^

**Figure 3:**
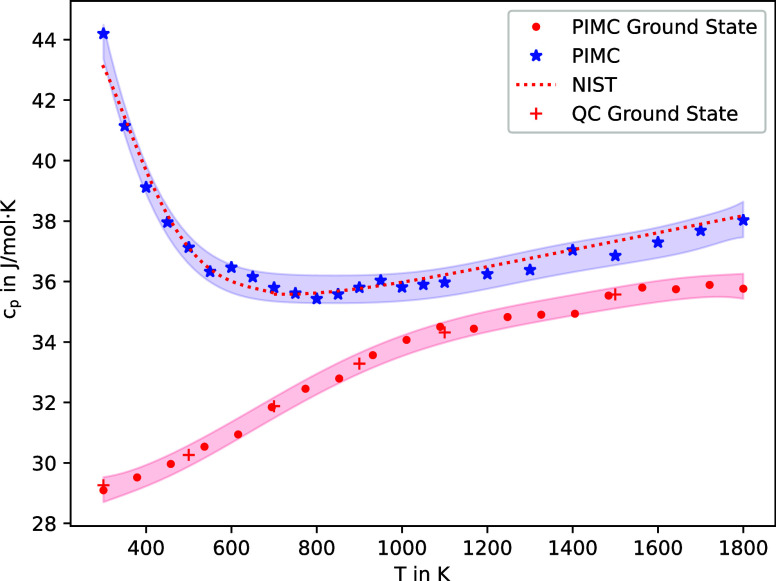
Heat capacity *c*_p_ of C_2_ as a function of temperature. The shaded areas give the standard error of *c*_p_ as computed via the PIMC algorithm by resampling.

For the simulation, each atom of the dimer was discretized into 20 beads and subsequently sampled for 10^9^ MC sweeps. For each MC sweep, a staging move, with a stage length of α = 12, or a CoM move, followed by a global excitation/relaxation to another electronic state was attempted. Due to the uncoupled nature of the electronic states, PoE moves are not necessary to compute as all beads need to be located at the same PES and thus computational resources can be saved. Figure 3 shows the resulting heat capacity *c*_p_ when considering both the *X*^1^Σ_g_^+^ ground state together with the first excited state *a*^3^Π*_u_* (PIMC), the *X*^1^Σ_g_^+^ ground state only (PIMC Ground State), and quantum chemically calculated heat capacities using the *X*^1^Σ_g_^+^ ground state (QC Ground State) at the CCSD/aug-cc-pVQZ level of theory^28^ in comparison to data from the NIST database (NIST).^37^ For the quantum mechanical calculation, the heat capacity is computed from translational, rotational (both within equipartition theorem), and vibrational contributions (harmonic oscillator approximation) while electronic contributions are neglected.^38^

Here, the importance of considering both electronic states becomes immediately evident. Especially at lower temperatures (400–1000 K), the possibility of occupying both electronic states has a significant effect on the heat capacity. This can be explained by the fact that at temperatures up to roughly 700 K, the energy becomes sufficient to thermally populate the higher-lying *a*^3^Π*_u_* state, leading to a temporary reduction of the heat capacity. This effect naturally cannot be captured by standard PIMC simulations where it is assumed that only the electronic ground state is populated.

## Conclusions

In this work, we presented a generalized approach to Path Integral Monte Carlo simulations that allows the calculation of static equilibrium properties of molecules at finite temperatures in which multiple low-lying potential energy surfaces are present and thermally accessible. Subsequently, we used it to obtain numerically exact results in both adiabatic and diabatic limits for a simple model system with harmonic adiabats and off-diagonal Gaussian-type coupling. The diabatic limit is particularly interesting for molecules containing transition metals like FeH^+^ or Fe^+^(H_2_O), which we previously investigated due to their possible astrochemical importance.^39,40^ Here, due to the complex electronic structure of the transition metal, a vast amount of energetically close and low-lying coupled PESs are present. With the proposed approach, such molecular systems could be investigated, considering both multiple potential energy surfaces and nonadiabatic effects.

Further, we showed that the adiabatic and diabatic results mainly differ in regions where a strong coupling between the PESs is present. This indicates that for molecular systems where nonadiabatic effects should be included, PoE moves only need to be computed if the beads are predominantly located in configurations that are concentrated in regions with strong couplings, making the approach also practically applicable to higher-dimensional PESs and systems with many electronic states.

Finally, two distinct molecules including a single and two PESs were investigated. For both H_2_ and C_2_, we could reproduce exact static finite temperature properties within the variance of the MC simulation, and for C_2_, we could illustrate nicely how at finite temperatures, the possibility of occupying multiple PESs might have a significant influence on the thermodynamic properties of molecules.

While the theoretical framework of this generalized approach to Path Integral Monte Carlo simulations itself is not fully novel,^14,15^ to our best knowledge, this is the first application to molecular systems and opens the possibility of exact simulations of molecular thermodynamic properties in molecules with multiple low-lying electronic states, in which one cannot assume that the nuclei evolve on a single potential energy surface. However, to do so, further developments will be needed. In particular, modeling the PESs of molecules including multiple atoms is not trivial as the underlying computational complexity scales exponentially, making it difficult to tackle with standard approaches. Here, it will be particularly interesting to explore the possibility of using modern deep learning techniques in combination with for example permutation invariant polynomial neural network^41^ to find a global representation of the PESs while avoiding exponential complexity. One further issue to explore is the efficiency of the proposed algorithm compared to the PIMD ansatz as staging PIMD was shown to be as efficient as PIMC.^42,43^ In the PIMC approach, the need to take many steps to ensure convergence could be possibly compensated by the flexibility in the choice of move types, which could be advantageous in larger systems.
